# Conservation of Mannan Synthesis in Fungi of the Zygomycota and Ascomycota Reveals a Broad Diagnostic Target

**DOI:** 10.1128/mSphere.00094-18

**Published:** 2018-05-02

**Authors:** Amanda R. Burnham-Marusich, Breeana Hubbard, Alexander J. Kvam, Marcellene Gates-Hollingsworth, Heather R. Green, Eric Soukup, Andrew H. Limper, Thomas R. Kozel

**Affiliations:** a Department of Microbiology and Immunology, University of Nevada, Reno School of Medicine, Reno, Nevada, USA; b Mayo Clinic, Rochester, Minnesota, USA; Carnegie Mellon University

**Keywords:** Mnn9, diagnostics, immunodetection, invasive fungal infection, lateral flow immunoassay, mannan, point of care

## Abstract

Ascomycetes and zygomycetes account for the majority of (i) fungi responsible for cutaneous, subcutaneous, and invasive human fungal infections, (ii) plant fungal pathogens, (iii) fungi that threaten global biodiversity, (iv) fungal agents of agricultural spoilage, and (v) fungi in water-damaged buildings. Rapid recognition of fungal infection (or contamination) enables early treatment (or remediation). A bioinformatics search found homologues of Saccharomyces cerevisiae Mnn9p present in members of the Zygomycota and Ascomycota phyla and absent in members of the Chytridiomycota and Basidiomycota. Mnn9p is a component of the yeast mannan polymerization complex and is necessary for α-1,6 mannan production. A monoclonal antibody (2DA6) was produced that was reactive with purified mannans of *Mucor*, *Rhizopus*, *Aspergillus*, *Fusarium*, and *Candida* species. Experimentation using a 2DA6 antigen capture enzyme-linked immunosorbent assay (ELISA) and extracts of fungi from the four phyla found agreement between the presence or absence of Mnn9p homologues and production or lack of production of mannan reactive with 2DA6. Studies of cell extracts from yeast mannan mutants identified α-1,6 mannan as the epitope recognized by 2DA6. To translate this finding into a point-of-use diagnostic, a 2DA6 lateral flow immunoassay was constructed that detected mannan in (i) extracts of dermatophytes and fungi that produce trauma-related infection and (ii) tissue from plants infected with Grosmannia clavigera or Sclerotium cepivorum. These studies (i) revealed that the conservation of α-1,6-linked mannan in fungi of the Zygomycota and Ascomycota can be exploited as a broad diagnostic target and (ii) have provided a means to detect that target in an immunoassay platform that is well suited for clinic or field use.

**IMPORTANCE** A key question asked when faced with an infection, an infestation, or environmental damage is whether it is a fungus. Identification of fungi as the cause of the problem can lead to remediation or treatment. Zygomycetes and ascomycetes account for the vast majority of fungal causes of human, animal, and plant disease, large-scale biodiversity loss, agricultural spoilage, and contamination of water-damaged buildings. These studies revealed the conservation of a common cell wall structural component of zygomycetes and ascomycetes to be a diagnostic target applicable to multiple pathogenic fungi and have leveraged that insight for practical use. Monoclonal antibodies reactive with this pan-fungal structure were produced and used to construct immunoassays (including ELISA and lateral flow assay) for detection of a broad range of pathogenic fungi.

## Abstract (precis)

A key question asked when faced with an infection, an infestation, or environmental damage is whether it is a fungus. Identification of fungi as the cause of the problem can lead to remediation or treatment. Zygomycetes and ascomycetes account for the vast majority of fungal causes of human, animal, and plant disease, large-scale biodiversity loss, agricultural spoilage, and contamination of water-damaged buildings. These studies revealed the conservation of a common cell wall structural component of zygomycetes and ascomycetes to be a diagnostic target applicable to multiple pathogenic fungi and have leveraged that insight for practical use. Monoclonal antibodies reactive with this pan-fungal structure were produced and used to construct immunoassays (including ELISA and lateral flow assay) for detection of a broad range of pathogenic fungi.

## INTRODUCTION

Fungi are a global threat to human, animal, plant, and environmental health. Fungal skin diseases affect 14% of the global population—approximately 1 billion people (1). Vulvovaginal candidiasis affects 70% to 75% of women at least once during their lives (2). Invasive fungal infections kill about 1.5 million people every year; most deaths are due to *Cryptococcus*, *Candida*, *Aspergillus*, and *Pneumocystis* spp. (3). Fungal infections are also producing biodiversity loss at a global scale; examples include the loss of some species of North American bats due to infection by the ascomycete fungus Pseudogymnoascus destructans and of many amphibian species worldwide due to infection by the chytrid fungus Batrachochytrium dendrobatidis (4). Fungi have been estimated to cause 72% of all disease-driven extinction/extirpation of animal species and 57% of all disease-driven plant species extinctions/extirpations (4). Finally, fungal diseases are a major threat to food security (4) and a serious health concern in water-damaged buildings (5).

An essential element of any strategy to control fungal disease is the need to rapidly diagnose infection. The combination of early diagnosis and timely use of antifungal agents mitigates the direct impact of infection, prevents the spread of disease, reduces opportunities for development of antifungal resistance, and controls costs. Current culture-based approaches to diagnosis may take days to produce a result. Molecular diagnostics take hours to produce a result and are often available only in specialized laboratories.

Immunodetection of fungal antigens is one approach to rapid diagnosis of fungal infection (reviewed in reference 6). For example, the Cr Ag lateral flow immunoassay for the capsular antigen of Cryptococcus neoformans is now in widespread use for diagnosis of cryptococcal meningitis in symptomatic patients and for prediction of risk for disease in asymptomatic patients (reviewed in reference 7). In another example, an immunoassay for *Aspergillus* galactomannan is a valuable aid for diagnosis of invasive aspergillosis (8).

A promising biomarker for detection of fungal infection is the mannoprotein located in fungal cell walls. Immunoassays for fungal mannans or galactomannans have been described previously for diagnosis of several invasive fungal disease, including candidiasis (9), aspergillosis (10), and histoplasmosis (11). The structures of cell wall mannoproteins are best described for the ascomycete yeasts Saccharomyces cerevisiae and *Candida* spp., where proteins are decorated with both *N*- and *O*-linked glycans. *O*-Linked oligosaccharides consist of one to five mannose units linked to serines or threonines (12). *N*-Linked glycans have an α-1,6-linked mannan chain of up to 50 mannose residues that extends from the *N*-glycan core. There are shorter side chains of α-1,2-linked mannose residues that terminate in α-1,3-linked mannose residues (13–15). Altogether, the *N*-linked yeast mannan is a highly branched structure with as many as 200 mannose residues. Other fungi such as the ascomycetes *Histoplasma* spp. and *Aspergillus* spp. produce galactomannans that have backbones that include α-1,6-linked mannose and that are heavily modified with side chains, which include galactose residues (16, 17).

The goal of this study was to identify epitopes of fungal mannans that are shared across the various fungi and to produce a monoclonal antibody (MAb) that could serve as a recognition reagent for a “pan-fungal” immunoassay. The results showed that the α-1,6 mannan backbone contains an epitope that is shared across the Ascomycota and Zygomycota phyla. A MAb that is reactive with this epitope was used to construct an immunoassay that is reactive with a broad range of pathogenic fungi that produce human disease or plant disease or that threaten biodiversity.

## RESULTS

### Identification of a MAb with broad reactivity across fungal mannans.

Mice were immunized with an Aspergillus fumigatus cellular antigen in an effort to produce MAbs that were reactive with fungal mannan. Splenocytes were harvested from mice with high levels of anti-mannan antibodies, and hybridomas were prepared. Numerous colonies were found that secreted antibodies that were reactive with purified A. fumigatus galactomannan. All positive colonies were given a second screen to assess the extent of reactivity of MAbs with purified mannans from other fungi, i.e., Mucor circinelloides, Fusarium solani, and Candida albicans. A range of cross-reactivity patterns was observed among the different hybridomas (Table 1). Some MAbs were reactive only with A. fumigatus galactomannan. Other MAbs were reactive with two or more of the different mannans. Two MAbs were reactive with mannan of all four fungi, suggesting pan-fungal reactivity. MAb 2DA6 was chosen for further evaluation and immunoassay construction based on (i) its strong binding across mannans of different fungal genera, (ii) robust growth and MAb production in cell culture, and (iii) production of antibody of the IgG1 subclass. The IgG1 subclass is typically easy to isolate from hybridoma supernatant fluid and shows no tendency for self-association that might produce background in immunoassays.

**TABLE 1:** IgG subclass and spectrum of mannan reactivity of MAbs produced in response to immunization with A. fumigatus cellular antigen

Mannan source	Reactivity of hybridoma cell line (IgG subclass)										
	4EE9 (IgG1)	1AG7 (IgG2b)	1AC1 (IgG1)	1CD6 (IgG2b)	3AE6 (IgG2b)	2BG2 (IgG2b)	2AG9 (IgG3)	4AF11 (IgG1)	3ED9 (IgG2b)	1AD7 (IgG1)	2DA6 (IgG1)
A. fumigatus	+	+	+	+	+	+	+	+	+	+	+
*Mucor* spp.	−	−	−	−	+	−	+	+	+	+	+
*Fusarium* spp.	−	−	+	+	−	+	+	+	−	+	+
C. albicans	−	−	−	−	−	−	−	−	+	+	+

An initial experiment was done to determine the extent to which MAb 2DA6 was reactive with purified mannans from different fungi. Mannans were isolated from *Mucor*, *Aspergillus*, *Fusarium*, and *Candida* spp. These fungal mannans were chosen for study because the composition of the mannans reflected the diversity of mannan structure, i.e., fucomannan (Mucorales [18]), galactomannan (*Aspergillus* [17]), and mannan (*Candida* [19]). Before the study, the glycosyl content of mannans isolated from each of the different fungi was assessed. In every case, the composition was consistent with the expected composition, i.e., mannan, galactomannan, or fucomannan (Table 2).

**TABLE 2:** Glycosyl composition of purified mannans

Fungus	Mannose (%)	Galactose (%)	Fucose (%)	Other sugars
*Mucor* spp.	57	2.4	41	Trace
C. albicans	99	1.2	None	None
*Fusarium* spp.	88	12	None	None
A. fumigatus	92	8.5	None	None

**FIG 1:**
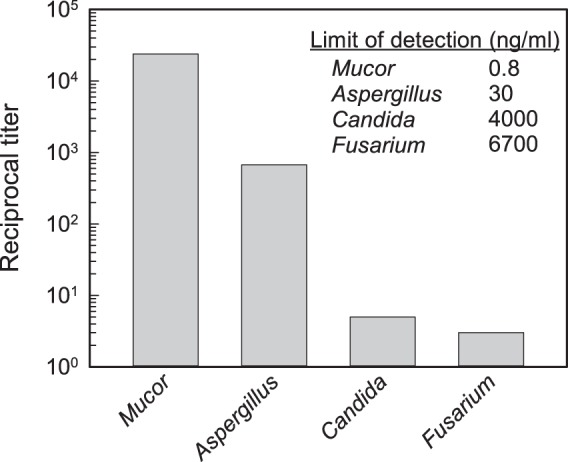
Reactivity of MAb 2DA6 with purified mannans of different fungal genera. Results are shown from a sandwich ELISA in which plates were (i) coated with MAb 2DA6 to enable mannan capture, (ii) incubated with serial dilutions of purified mannan (20 µg/ml starting concentration), and (iii) incubated with HRPO-labeled MAb 2DA6. (Inset) Limit of detection (in nanograms per milliliter) of the sandwich ELISA for mannans isolated from different fungal genera.

MAb binding was evaluated by use of a sandwich enzyme-linked immunosorbent assay (ELISA) in which microtiter plates were first coated with unlabeled MAb 2DA6. The wells were then incubated with various amounts of each mannan. Capture of the mannans was determined by use of enzyme-linked (horseradish peroxidase [HRPO]) MAb 2DA6. The results (Fig. 1) showed that each of the mannans was captured in the sandwich ELISA. However, the sensitivity of the sandwich ELISA for detection of the mannans was highly variable, with the following order of relative sensitivities: *Mucor* > *Aspergillus* > *Candida* > *Fusarium*.

### α-1,6-linked mannose in the mannan backbone is required for MAb 2DA6 binding.

A common structural feature of mannans of the Mucorales, *Aspergillus*, and *Candida* species is the presence of α-1,6-linked mannose residues in the backbone (17–19). The presence of this common backbone structure, despite considerable variability in side chain structure, suggested that a component of the mannan backbone is the epitope that is recognized by MAb 2DA6.

There is an extensive set of S. cerevisiae mannosylation mutants that would allow evaluation of the contribution of various structural elements of yeast mannan to binding by MAb 2DA6. Specifically, Mnn9p is a component of mannan polymerase complex I (M-Pol I) and M-Pol II, which are required for extension of the α-1,6-mannan backbone (20). *Mnn9* mutants produce a highly truncated α-1,6-mannan backbone. Mnn2p attaches the initial α-1,2-mannose unit that branches off the α-1,6-mannan backbone (21, 22). *Mnn2* mutants produce an unbranched α-1,6-mannan chain that is capped with a single α-1,2-linked mannose (23–25).

**FIG 2:**
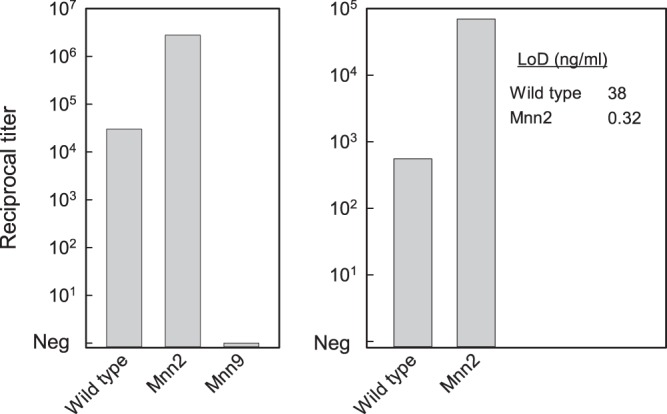
Reactivity of MAb 2DA6 with mannans of the wild-type and the indicated mannan mutants of S. cerevisiae. (Left) A sandwich ELISA constructed from MAb 2DA6 was used to assess reactivity of cell extracts from the wild-type and *Mnn2* and *Mnn9* mutants. (Right) Reactivity of purified wild-type and *Mnn2* mutant mannans in the sandwich ELISA. The starting concentration for the purified mannans was 20 µg/ml. Neg, negative. (Inset) Limit of detection (LoD [in nanograms per milliliter]) of the sandwich ELISA for mannans isolated from the wild-type and *Mnn2* mutant strains.

Hot citrate extracts were prepared from the parental S. cerevisiae BY4743 strain (mannan produced by BY4743 is termed wild type for the purposes of this report) and from the *Mnn2* and *Mnn9* mutants. Extracts were evaluated using the sandwich ELISA constructed from MAb 2DA6. The results showed no reactivity with extracts from the *Mnn9* mutant strain. In contrast, there was a 93-fold increase in the titer of extract from the *Mnn2* mutant compared to extract from the wild-type strain (Fig. 2—left).

The difference in titers between the extracts from the *Mnn2* mutant and those from the wild-type strain could have been due to intrinsic differences in the ability of the mannans to be captured in the sandwich ELISA or to differences in production or extractability of mannan from the yeast cells. As a consequence, mannan was purified from hot citrate extracts of the *Mnn2* mutant and the wild-type strain. Examination of the reactivity of the two purified mannans in the sandwich ELISA showed that there was a 120-fold-higher titer for mannan from the *Mnn2* mutant than for wild-type mannan from the wild type (Fig. 2—right). Indeed, sandwich ELISA showed a greater sensitivity for detection for *Mnn2* mutant mannan (limit of detection = 0.32 ng/ml) than for mannans of all other fungi examined in the experiments whose results are presented in Fig. 1. Thus, in this assay, increases in titer reflect increased reactivity of MAb 2DA6 with the sample, likely through increased access to the antibody’s epitope.

**FIG 3:**
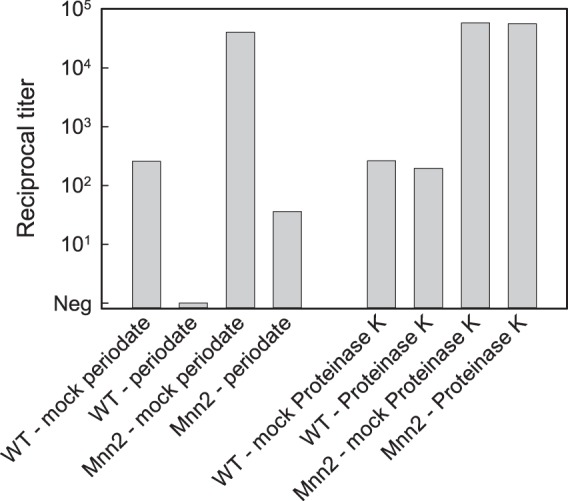
Effect of treatment of wild-type (WT) and Mnn2 mutant mannan with periodate and proteinase K on reactivity with MAb 2DA6 in a sandwich ELISA. Mannans were treated with each reagent or were subjected to a mock treatment where all reagents and reactions were identical to those used with the treated group but where the periodate or proteinase K was omitted. The starting concentration for the mannans was 20 µg/ml.

Yeast mannans are components of cell wall glycoproteins that are modified with both *N*-linked and *O*-linked glycans. This raises an issue as to whether MAb 2DA6 binds the carbohydrate or protein components. Mild periodate oxidation cleaves carbohydrate vicinal hydroxyl groups without altering the structure of polypeptide chains (26–28). Treatment of both wild-type and *Mnn2* mutant yeast mannan with periodate led to a >99% loss of reactivity with MAb 2DA6 in the sandwich ELISA constructed from MAb 2DA6 (Fig. 3). In contrast, treatment of the two mannanoproteins with proteinase K had no detectable effect on reactivity of either mannan with MAb 2DA6 in the sandwich ELISA (Fig. 3).

### Reactivity of MAb 2DA6 across fungal phyla—bioinformatics analysis and direct experimentation.

Because Mnn9p is a key component of both α-1,6-mannosyltransferase complexes in S. cerevisiae, and because MAb 2DA6 recognizes yeast mannan in a *Mnn9*-dependent manner (Fig. 2), we reasoned that other fungal species that contained a protein sequence(s) with significant homology to Mnn9p from S. cerevisiae would also produce mannan that is reactive with MAb 2DA6. We performed a BLASTP analysis for several fungal species of interest using S. cerevisiae Mnn9p as the query to search the NCBInr protein database, which includes all nonredundant GenBank coding sequence (CDS) translations as well as all PDB, Swiss-Prot, PIR, and PRF sequences (Table 3). Alignments with expected values of less than 1e−30 were considered significant. The species of fungi that were evaluated included one member of the Chytridiomycota, two zygomycetes, three basidiomycetes, and 14 ascomycetes, including *Pneumocystis* spp. The results (Table 3) showed no homologues among any of the chytridiomycetes or basidiomycetes. In contrast, there were S. cerevisiae Mnn9p homologues in both of the zygomycetes (*Rhizopus* and *Mucor*) and in 11 of the 12 ascomycetes for which there were enough sequences for analysis. *Pneumocystis* spp. represented the only ascomycete species with a sequenced genome that did not have a Mnn9p homologue.

**TABLE 3:** Relationship between fungal taxonomy and production of the MAb 2DA6 epitope

Fungus	Disease	Mnn9p homology^a^	MAb 2DA6 reactivity with cell extract^b^
Chytridiomycota			
Batrachochytrium dendrobatidis	Chytridiomycosis in amphibians	None	No
			
Zygomycota			
Rhizopus oryzae	Mucormycosis	4e−70^c^	Yes
*Mucor* spp.	Mucormycosis	9e−69^c^	Yes
			
Basidiomycota			
Cryptococcus neoformans	Cryptococcosis	None	No
Ustilago maydis	Corn smut	None	No
Malassezia furfur	Pityriasis versicolor	None^c^	No
			
Ascomycota			
*Pneumocystis* spp.	Pneumocystis pneumonia	None^c^	No
Schizosaccharomyces pombe	Fission yeast—not a pathogen	6e−111	Yes
Pseudogymnoascus destructans	Bat white-nose syndrome	3e−118	Yes
Microsporum canis	Dermatophytosis	4e−115	Yes
Trichophyton rubrum	Dermatophytosis	4e−122	Yes
Epidermophyton floccosum	Dermatophytosis	ND^d^	Yes
Aspergillus fumigatus	Invasive aspergillosis	5e−121	Yes
Talaromyces marneffei	Penicilliosis	1e−120	Yes
Botrytis cinerea	Grey rot and noble rot in plants	6e−113	Yes
Sclerotium cepivorum	White rot in *Allium* species	ND	Yes
Fusarium solani species complex	Sea turtle hatch failure; fungal keratitis; fusariosis	3e−121	Yes
Scedosporium apiospermum	Scedosporiosis; mycetoma	5e−115	Yes
Magnaporthe oryzae	Rice blast disease	2e−119	Yes
Saccharomyces cerevisiae	Not normally a pathogen	NA^e^	Yes
Candida albicans	Invasive and mucosal candidiasis	1e−149	Yes

^a^ The values indicated represent BLASTP expected values; <1e−30 was considered significant.

^b^ Reactivity determined by antigen capture ELISA. Results shown are summarized from Fig. 4.

^c^ Data represent results of BLASTP analysis of all NCBInr sequences from the indicated genus.

^d^ ND, not done (too few sequences in NCBI database for homology search).

^e^ NA, not applicable (S. cerevisiae was the Mnn9 sequence source for all homology testing).

**FIG 4:**
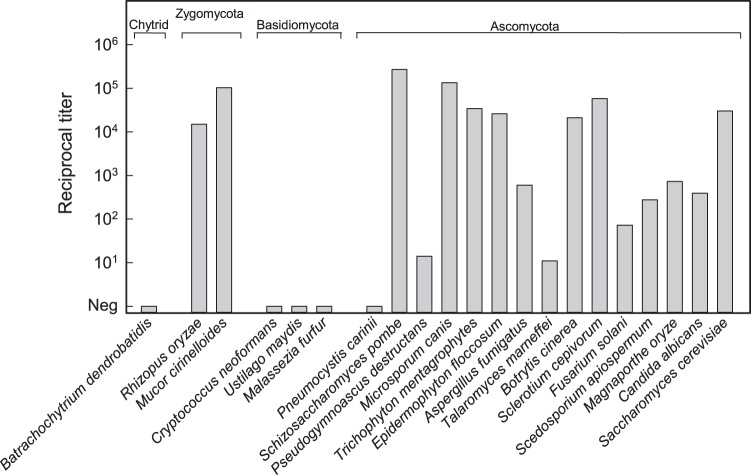
Reactivity of hot citrate extracts from various fungi in a sandwich ELISA constructed from MAb 2DA6 Pneumocystis carinii isolated from infected rat lung was used for that fungus. In all other cases, extracts were prepared from mycelia or yeasts from culture.

Direct experimentation was done to validate the *in silico* predictions of MAb 2DA6 reactivity. Hot citrate extracts were prepared from cultures of most of the fungal species listed in Table 4, including the chytridiomycete Batrachochytrium dendrobatidis, two zygomycetes, three basidiomycetes, and 14 members of the Ascomycota phylum. In the case of *Pneumocystis* spp., extracts were prepared from organisms purified from infected rat lung. The extracts were evaluated for reactivity in the sandwich ELISA constructed from MAb 2DA6. The results (Fig. 4) showed complete agreement between the experimental results and results predicted from the bioinformatics analysis for the presence of Mnn9p homologues. Specifically, extracts from the B. dendrobatidis isolate and three different members of the Basidiomycota (Cryptococcus neoformans, Ustilago maydis, and *Malassezia furfur*) failed to react in the sandwich ELISA, which was as predicted. In contrast, extracts from both fungi of the Zygomycota (*Rhizopus* and *Mucor*) were highly reactive, also as predicted. Finally, 14 of 15 extracts from the Ascomycota were reactive. Notably, extract from P. carinii purified from lung of infected rats was negative, which was consistent with the bioinformatics-based prediction.

**TABLE 4:** Sources of cultures used for study and growth conditions

Fungus (mannan component)	Strain	Source^a^	Growth condition	
			Medium	Temp
Aspergillus fumigatus	ATCC MYA-4609	ATCC	RPMI 1640, 2% glucose	30°C
Batrachochytrium dendrobatidis	CJB5	J. Voyles	TGhL medium^b^	RT^c^
Botrytis cinerea	B05.10	FGSC	RPMI 1640, 2% glucose	25°C
Candida albicans	ATCC MYA-2876	ATCC	RPMI 1640, 2% glucose,	30°C
Cryptococcus neoformans	602	T. Kozel	RPMI 1640, 2% glucose	30°C
Epidermophyton floccosum	ATCC 38486	ATCC	RPMI 1640, 2% glucose	30°C
Fusarium falciforme	ATCC MYA-3636	ATCC	RPMI 1640, 2% glucose	30°C
Magnaporthe oryzae	ATCC 201236	ATCC	RPMI 1640, 2% glucose	25°C
*Malassezia furfur*	ATCC 14521	ATCC	RPMI 1640, 2% glucose, 0.1 mg/ml Tween 80	30°C
Microsporum canis	ATCC 36299	Fisher Scientific	RPMI 1640, 2% glucose	26°C
Mucor circinelloides	ATCC 8542	ATCC	RPMI 1640, 2% glucose	30°C
Talaromyces marneffei	ATCC 18224	ATCC	RPMI 1640, 2% glucose	25°C
Pseudogymnoascus destructans	ATCC MYA-4855	ATCC	RPMI 1640, 2% glucose	4–6°C
Rhizopus oryzae	ATCC 11886	ATCC	RPMI 1640, 2% glucose	30°C
Saccharomyces cerevisiae	BY4743	W. Courchesne	RPMI 1640, 2% glucose, 40 µg/ml uridine	30°C
S. cerevisiae (Mnn2)	Clone ID 33152	GE Dharmacon	RPMI 1640, 2% glucose, 40 µg/ml uridine	30°C
S. cerevisiae (Mnn9)	Clone ID 32778	GE Dharmacon	RPMI 1640, 2% glucose, 40 µg/ml uridine	30°C
Scedosporium apiospermum	ATCC MYA-3635	ATCC	RPMI 1640, 2% glucose	RT
Schizosaccharomyces pombe	ATCC 14548	ATCC	RPMI 1640, 2% glucose	30°C
Sclerotium cepivorum		S. Wang	RPMI 1640, 2% glucose	RT
Trichophyton rubrum	ATCC MYA-4438	ATCC	RPMI 1640, 2% glucose	RT
Ustilago maydis	ATCC MYA-4924	ATCC	RPMI 1640, 2% glucose	25°C

^a^ Abbreviation or affiliation: ATCC, American Type Culture Collection; J. Voyles, University of Nevada—Reno; FGSC, Fungal Genetics Stock Center, Kansas State University; T. Kozel, University of Nevada—Reno; W. Courchesne, University of Nevada—Reno; S. Wang, Nevada Department of Agriculture; ID, identifier.

^b^ TGhL, tryptone gelatin hydrolysate lactose.

^c^ RT, room temperature.

Fungi pose threats to animal, human, agricultural, and environmental health. As a consequence, a bioinformatics search for Mnn9p homologues was done to predict the likely reactivity of MAb 2DA6 with common fungal threats to global well-being (Tables S1 to S7). The results show probable reactivity with 7/10 of the major causes of plant pathology (see Table S1 in the supplemental material), 8/10 of the fungi causing major invasive human fungal infections (Table S2), 9/10 of the fungi causing major cutaneous and subcutaneous fungal infections (Table S3), 4/6 examples of fungi responsible for large-scale biodiversity loss (Table S4), 5/7 of the fungi causing fungal-driven extinction and extirpation events in plants and animals (Table S5), 6/7 fungal agents of food and agricultural spoilage (Table S6), and 8/10 fungi commonly found in water-damaged building materials (Table S7).

10.1128/mSphere.00094-18.1 TABLE S1  Predicted reactivity of MAb 2DA6 with major fungal pathogens in plant pathology. Download TABLE S1, PDF file, 0.03 MB. Copyright © 2018 Burnham-Marusich et al. 2018 Burnham-Marusich et al. This content is distributed under the terms of the Creative Commons Attribution 4.0 International license.

10.1128/mSphere.00094-18.2 TABLE S2  Predicted reactivity of MAb 2DA6 with common fungal causes of invasive fungal infection in humans. Download TABLE S2, PDF file, 0.03 MB. Copyright © 2018 Burnham-Marusich et al. 2018 Burnham-Marusich et al. This content is distributed under the terms of the Creative Commons Attribution 4.0 International license.

10.1128/mSphere.00094-18.3 TABLE S3  Predicted reactivity of MAb 2DA6 with significant fungal causes of skin, hair, nail, eye, and cutaneous and subcutaneous fungal infections in humans. Download TABLE S3, PDF file, 0.03 MB. Copyright © 2018 Burnham-Marusich et al. 2018 Burnham-Marusich et al. This content is distributed under the terms of the Creative Commons Attribution 4.0 International license.

10.1128/mSphere.00094-18.4 TABLE S4  Predicted reactivity of MAb 2DA6 with major fungal causes of potential biodiversity loss. Download TABLE S4, PDF file, 0.03 MB. Copyright © 2018 Burnham-Marusich et al. 2018 Burnham-Marusich et al. This content is distributed under the terms of the Creative Commons Attribution 4.0 International license.

10.1128/mSphere.00094-18.5 TABLE S5  Predicted reactivity of MAb 2DA6 with agents of fungal disease-driven and regional extirpation events across animal and plant taxa. Download TABLE S5, PDF file, 0.03 MB. Copyright © 2018 Burnham-Marusich et al. 2018 Burnham-Marusich et al. This content is distributed under the terms of the Creative Commons Attribution 4.0 International license.

10.1128/mSphere.00094-18.6 TABLE S6  Predicted reactivity of MAb 2DA6 with selected fungal agents of food and agricultural spoilage. Download TABLE S6, PDF file, 0.03 MB. Copyright © 2018 Burnham-Marusich et al. 2018 Burnham-Marusich et al. This content is distributed under the terms of the Creative Commons Attribution 4.0 International license.

10.1128/mSphere.00094-18.7 TABLE S7  Predicted reactivity of MAb 2DA6 with fungal genera commonly found on water-damaged building materials. Download TABLE S7, PDF file, 0.03 MB. Copyright © 2018 Burnham-Marusich et al. 2018 Burnham-Marusich et al. This content is distributed under the terms of the Creative Commons Attribution 4.0 International license.

### Lateral flow immunoassay for detection of fungal mannan.

The sandwich ELISA format used for the studies whose results are shown in Fig. 1 to 4 has the advantage that it produces quantitative results representing high sensitivity. However, the ELISA format takes several hours to complete and requires skilled laboratory personnel and considerable laboratory infrastructure. In contrast, the lateral flow immunoassay (LFIA) platform produces a rapid (<15-min) result and is well suited to use at the point of need. Therefore, an LFIA was constructed from MAb 2DA6 and was used to assay mannans in extracts from selected fungi that were predicted to have Mnn9p homologues and were shown by experimental results to be reactive in the sandwich ELISA constructed from MAb 2DA6 (Fig. 4).

**FIG 5:**
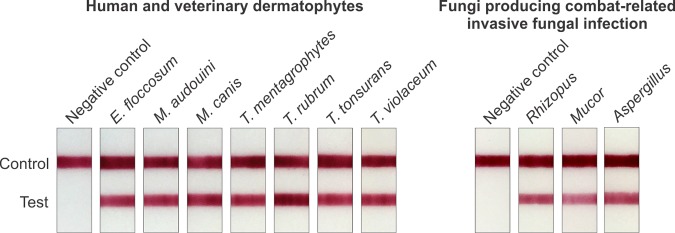
Detection of mannan in hot citrate extracts from cultures of medically relevant fungi in a lateral flow immunoassay constructed from MAb 2DA6. (Left) Extracts from cultures of *Epidermophyton* spp., *Microsporum* spp., and *Trichophyton* spp. that cause dermatophyte infection in humans and animals. (Right) Extracts from cultures of fungi that produce combat-related invasive fungal infection. Negative control, citrate buffer.

In one example, hot citrate extracts were prepared from cultures of seven fungi that produce dermatophyte infection in humans and animals. The results showed high levels of reactivity that were similar across the various dermatophytes (Fig. 5—left). In another example, extracts were prepared from cultures of the most common fungi producing trauma-related invasive fungal infection (IFI), i.e., members of the order Mucorales and *Aspergillus* spp. (29, 30). Mannan was readily detectable by LFIA analysis of extracts from all of the IFI fungi tested (Fig. 5—right).

**FIG 6:**
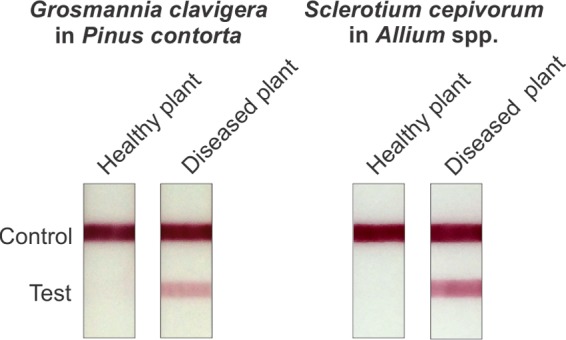
Use of LFIA constructed from MAb 2DA6 to detect mannan in hot citrate extracts from tissue of Pinus contorta (lodgepole pine) infected with Grosmannia clavigera (blue stain fungus [left]) and tissue from *Allium* species (onion) infected with Sclerotium cepivorum (Allium white rot [right]). Results are shown for extracts from healthy and diseased plants.

The LFIA also detected fungal mannan in extracts from infected plant tissue. In one example, hot citrate extracts were prepared from healthy Allium cepa (common onion) or A. cepa infected with *Allium* white rot (Sclerotium cepivorum). In another example, an extract was prepared from healthy Pinus contorta (lodgepole pine) or P. contorta infected with blue stain fungus (Grosmannia clavigera). The results consisted of a positive reaction using tissue from infected plants and no reaction using tissue from healthy plants (Fig. 6).

## DISCUSSION

Immunoassays that detect microbial antigens are used for diagnosis of many infectious diseases. Examples include cryptococcal meningitis; invasive aspergillosis; infections by group A *Streptococcus*, influenza virus, and respiratory syncytial virus; and chlamydia. In the present study, we produced a MAb that can address the issue of whether a given isolate represents a fungus. Our results indicate that the test detects the presence of fungi of the Zygomycota and Ascomycota that produce a Mnn9p homologue but not the presence of fungi of the Chytridiomycota or Basidiomycota. Importantly, ascomycetes and zygomycetes account for almost all of the fungi responsible for invasive, cutaneous, and subcutaneous human fungal infections; most plant fungal pathogens; and most fungi that threaten global diversity (Tables S1–7).

All evidence indicates that the epitope recognized by MAb 2DA6 is located on the α-1,6 backbone of cell wall mannan. First, where structures have been published, MAb 2DA6 was shown to be reactive with purified mannans (Fig. 1) or cell extracts of fungi (Fig. 4 and 5) that (i) have cell wall mannans with a backbone comprised primarily or entirely of α-1,6 mannose (e.g., S. cerevisiae, C. albicans, *Mucor*, and *Rhizopus*) and the dermatophytes (18, 19, 25, 31–33) or (ii) have mannans where α-1,6 mannose is a significant component of the backbone (e.g., A. fumigatus) (17). Second, MAb 2DA6 was reactive with wild-type yeast mannan and mannan from a *Mnn2* mutant, but extracts of a *Mnn9* mutant were not reactive (Fig. 2). *Mnn2* mutants are unable to add the initial α-1,2-mannose unit that branches off the α-1,6-mannan backbone (21, 22). *Mnn2* mutants thus produce an unbranched α-1,6-mannan chain that is capped with a single α-1,2-linked mannose (23, 24). In contrast, Mnn9 mutants do not form a multiprotein complex with α-1,6 mannosyl transferase activity and are unable to form the long α-1,6-linked backbone of yeast mannan (20, 23). Finally, there was complete congruence between the presence of a Mnn9p homologue as determined by bioinformatics search (Table 3) and the presence of mannans in cell extracts that are reactive with MAb 2DA6 (Fig. 4).

Our survey of extracts from 21 fungi (Fig. 4) and of seven purified mannans (Fig. 1 and 2) showed considerable variability in the sensitivity for detection by MAb 2DA6, i.e., in the MAb 2DA6 assay limit of detection. One possible explanation for the differences in the reactivities of mannans in fungal extracts is variability in either the production or the extractability of mannans. However, the finding of similar patterns of variability in the reactivity of MAb 2DA6 with purified mannans suggests that structural differences likely explain the differences in sensitivity. This argument is supported by a comparative evaluation of the reactivity of MAb 2DA6 with wild-type and *Mnn2* mutant mannans (Fig. 2). Wild-type mannan has extensive branching from the α-1,6 mannan backbone by α-1,2 residues; *Mnn2* mutant mannan lacks such branching and consists entirely of a long α-1,6-linked backbone (25). The limit of detection for the sandwich ELISA for purified *Mnn2* mutant mannan was >100 times lower than the limit of detection for wild-type mannan (i.e., MAb 2DA6 has more than 100-fold-better sensitivity for the α-1,6 mannan backbone without α-1,2-linked side chain branches than for the wild-type α-1,6 mannan backbone with side chains). This result suggests that the α-1,2-linked mannose side chains from the backbone block MAb 2DA6 binding or reduce the amount of available binding sites. A similar result was reported by Raschke et al., who found that polyclonal antibodies raised against *Mnn2* mutant yeast failed to react with wild-type mannan (24).

The extent to which side chain blockade of antibody binding to the α-1,6 mannan backbone impacts the utility of the MAb 2DA6 immunoassay depends on the individual fungus and on the requirements for assay sensitivity. For example, extracts from *Rhizopus* and *Mucor* and the dermatophytes produced very high titers in the sandwich ELISA relative to the other fungi tested (Fig. 4), indicating that any side chains present in species from these genera had negligible blocking activity. In other instances, e.g., mannans of *Fusarium* or *Candida*, titers with the 2DA6 sandwich ELISA were lower, indicating that side chain blockade of MAb binding likely impacts the assay sensitivity with these fungi (Fig. 1 and 4). Requirements for assay sensitivity need to be determined on a case-by-case basis. For example, the concentration of C. albicans in swabs of vaginal candidiasis may be high (34), in which case, a clinically useful immunoassay is quite possible despite partial blockade of access of antibodies to the backbone.

An alternative approach to assay development for cases in which side chain blockade does present an issue for assay utility would be sample treatment to remove blocking of side chains. For example, Reiss noted that side chains on some fungal mannans are susceptible to hydrolysis at high pH (35). Alternatively, treatment of sample extracts with glycolytic enzymes could be used to increase immunoassay sensitivity for fungal mannans that show considerable levels of substitution by α-1,2-linked side chains on the α-1,6-linked mannan backbone. For example, Jones and Ballou produced an enzyme from *Arthrobacter* GJM-1 that was an exoglycosidase which removed α-1,2-linked and α-1,3-linked side chains from S. cerevisiae and C. albicans mannans to leave an α-1,6-linked backbone (36). In preliminary experiments, we have demonstrated the ability of a partially purified exoglycosidase from *Arthrobacter* GJM-1 to enhance reactivity of MAb 2DA6 with exoglycosidase-treated mannans of *Fusarium*, C. albicans, and S. cerevisiae.

The pan-fungal reactivity of immunoassays constructed from MAb 2DA6 across the Zygomycota and Ascomycota has both advantages and disadvantages for diagnosis of fungal infection. A major strength of a pan-fungal immunoassay is the ability to broadly determine the presence of fungal infection. A positive result would trigger an early antifungal intervention. A negative result would facilitate antifungal stewardship and spare individuals or the environment possible exposure to the side effects of many antifungal agents. The weakness of a pan-fungal immunoassay is the absence of genus or species information. In instances where there is only one fungus indicated in a differential diagnosis, identification of fungal infection without genus or species information would be sufficient for appropriate diagnosis and prompt treatment initiation. In other cases where there are multiple fungi indicated in a differential diagnosis, identification at the genus or species level might impact the choice of antifungal. Nevertheless, early recognition of fungal infection, regardless of genus or species, would enable empirical therapy pending results of culture.

Furthermore, although mannan comprised of α-1,6-mannose residues is considered a hallmark of the Fungi kingdom, α-1,6-mannose residues have also been documented in *Mycobacterium* spp. and Corynebacterium glutamicum (37–39). However, whereas each S. cerevisiae mannan polymer contains approximately 40 to 60 α-1,6-linked mannose residues, Mycobacterium smegmatis and Corynebacterium glutamicum generate much shorter α-1,6-linked mannose backbones of just 12 and 20 to 25 mannose residues, respectively (23, 37–40). In addition, the α-1,6-linked mannans produced in M. smegmatis and C. glutamicum also showed extensive substitutions with α-1,2-linked-mannan (37–40). Such substitutions may reduce their reactivity with MAb 2DA6 based on our data showing greater MAb 2DA6 reactivity with S. cerevisiae mutants lacking α-1,2-linked mannan substitutions than with wild-type S. cerevisiae (Fig. 2). As with development of all diagnostics, the differential diagnosis should be carefully considered for each disease or indication for which development of a MAb 2DA6-based diagnostic is pursued. Detailed experimental evaluations of the analytical and clinical specificity of MAb 2DA6 with a custom-tailored list of other potential pathogens for the differential diagnosis should be performed during each assay’s development, as is already standard practice for FDA clearance.

The ability to use bioinformatics searches for Mnn9p homologues to predict whether a given fungus might produce a mannan that is reactive with MAb 2DA6 greatly facilitates development of new applications for the 2DA6 pan-fungal immunoassay. One example of the potential utility of this approach is provided by bat white-nose syndrome, which is caused by the ascomycete Pseudogymnoascus destructans, which is currently causing catastrophic declines in the populations of multiple species of bats in eastern North America (41, 42). A bioinformatics search for Mnn9p homologues in P. destructans found a protein (NCBI accession no OAF58468.1) with a high degree of homology (3e−118) (Table 3; see also Table S4 in the supplemental material). This *in silico* analysis was followed by direct experimentation which showed that an extract from a P. destructans culture was reactive in a sandwich ELISA constructed from MAb 2DA6 (Fig. 4). In a similar manner, investigators can use bioinformatics analysis to predict potential success for use of the pan-fungal epitope as a biomarker for immunodetection of many fungi that threaten human, animal, plant, or biodiversity health (see, e.g., Tables S1 to S7).

The unusual case of *Pneumocystis* spp. further highlights the utility of a bioinformatics approach to predicting the reactivity of a given fungal pathogen with the MAb 2DA6 immunoassay. *Pneumocystis* spp. are ascomycetes and by phylogenetic comparison alone might be predicted to react with MAb 2DA6: 14 of 14 of the other ascomycetes tested reacted with the 2DA6 immunoassay (Fig. 4). However, our bioinformatics analysis of P. jirovecii, P. murina, and P. carinii indicated that these three species lack any sequences with significant homology to Mnn9p from S. cerevisiae. As predicted by this bioinformatics analysis, P. carinii extracts did not react with the 2DA6 immunoassay (Fig. 4). Unlike other ascomycetes, *Pneumocystis* spp. appear to be obligate pathogens, and they have undergone extensive gene loss during their adaptation to the mammalian lung environment (43). In addition to their lack of a Mnn9p homologue, *Pneumocystis* spp. also lack all of the other enzymes of mannan polymerase complexes I and II that are necessary for extension of the α-1,6-linked mannan backbone, and they even lack enzymes for chitin synthesis or degradation (43). The lack of a Mnn9p homologue in *Pneumocystis* spp. and the absence of MAb reactivity with *Pneumocystis* spp. likely reflect these adaptations of *Pneumocystis* spp. to their evolutionary niche as obligate pathogens.

Cases of convergent evolution, where a pathogen contains enzymes that are not homologous with S. cerevisiae Mnn9p and yet still have α-1,6-mannosyltransferase activity, represent a potential limitation to bioinformatics-based predictions of MAb 2DA6 reactivity. The reactivity of MAb 2DA6 in such cases would not be predictable by a homology-based BLASTP search but could be detected by empirical testing of MAb 2DA6 with the given pathogen.

In summary, an epitope found on the α-1,6 mannan backbone of fungal mannans is a diagnostic target for immunoassays that detect the presence of fungi of the Zygomycota and Ascomycota phyla. Fungi of the Chytridiomycota and Basidiomycota do not produce the reactive epitope. Bioinformatics analysis for production of Mnn9p, which is necessary for backbone synthesis, can be used to predict production of a mannan that is reactive with MAb 2DA6. Finally, immunoassays in ELISA and LFIA formats can detect mannan in extracts from fungal cultures and tissues from plants with infection by fungi having Mnn9p homologues.

## MATERIALS AND METHODS

### Fungal cultures and infected tissue.

Sources of all fungal cultures and conditions for growth are provided in Table 4. P. carinii was isolated from infected rat lung as described previously (44). Wood from Pinus contorta infected with blue stain fungus (Grosmannia clavigera) was provided by G. Blomquist, University of Nevada—Reno. Allium cepa (common onion) infected with *Allium* white rot (Sclerotium cepivorum) was provided by S. Wang, Nevada Department of Agriculture.

### Mannan isolation and purification from fungal cultures.

Mannan was isolated from cultures of A. fumigatus, C. albicans, F. solani, and M. circinelloides. The length of culture varied with each fungus, ranging from 48 h (C. albicans) to 7 days (M. circinelloides). Fungal cells were removed from culture medium by filtration through a 0.22-µm-pore-size filter (Nalgene 585-4520). The C. albicans culture required clarification by sedimentation. Yeast and mycelia mats were subjected to mechanical disruption using 425-to-600-µm-diameter glass beads (Sigma-Aldrich, St. Louis, MO). Sterile water was added to resuspend fungi and combined with an equal volume of glass beads. Mechanical disruption was performed for 2 min followed by 5 min of incubation on ice. Five rounds were completed before centrifugation was performed to remove cellular debris followed by filtration through a 0.22-µm-pore-size filter. The culture supernatant fluids and cell lysate supernatant fluids were pooled for each fungus (approximately 5 liters), passed through a 0.22-µm-pore-size filter, and concentrated to 100 ml with a Millipore Labscale tangential flow filtration system that was fitted with a Pellicon XL 50 cassette.

In the case of S. cerevisiae, mannan was extracted from cell pellets by the hot citrate method of Peat et al. (13). Mannan was purified from the supernatant fluid and cell lysate pool by affinity chromatography on concanavalin A-Sepharose 4B and elution with α-d-methylmannopyranoside. The concentration of purified mannan was determined by the phenol-sulfuric acid assay of Dubois (45), using glucose as a standard.

Mannan compositions were determined by the Complex Carbohydrate Research Center (University of Georgia, Athens, GA). Glycosyl composition analysis was performed by combined gas chromatography/mass spectrometry (GC/MS) assays of the per-*O*-trimethylsilyl (TMS) derivatives of the monosaccharide methyl glycosides, which were produced from each sample by acidic methanolysis.

### Extraction of crude mannan from fungal cultures and tissues of fungus-infected plants.

Mannan was extracted from intact fungal elements from culture or from tissues of infected or healthy plants by the hot citrate method of Peat et al. (13). Briefly, cells and tissue were washed with phosphate-buffered saline (PBS), resuspended in 10 volumes of 0.1 M citrate buffer (pH 7.0), and autoclaved for 45 min. The suspension was clarified by centrifugation followed by filtration through a 0.22 µM-pore-size filter and was frozen at −80 C.

### MAb production.

Immunization of mice for production of splenocytes was approved by the Institutional Animal Care and Use Committee of the University of Nevada—Reno. Mice from The Jackson Laboratory were hyperimmunized using an immunization schedule based on methods described in references 46 and 47. Briefly, A. fumigatus cells were inactivated using formalin, washed in PBS followed by water, bead-beaten, lyophilized, and resuspended in sterile PBS at 1 mg/ml (wt/vol). Mice were immunized with 100 µl of the A. fumigatus cell suspension via the intraperitoneal route every 2 days for a total of 10 injections. Mice were then rested for 4 weeks, after which they received another set of 10 immunizations. Splenocytes from the mice with the highest serum titers (>100,000) against purified A. fumigatus galactomannan by ELISA were isolated and cryopreserved as described previously (48). Hybridomas were generated from the cryopreserved splenocytes via standard protocols. All hybridoma wells were initially screened by ELISA for reactivity with purified A. fumigatus galactomannan in the solid phase. Hybridomas secreting antibody reactive with A. fumigatus galactomannan were expanded and rescreened for continued reactivity with A. fumigatus galactomannan as well as for reactivity with purified mannan or fucomannan from F. solani, C. albicans, and M. circinelloides spp. Hybridomas of interest were subjected to multiple rounds of cloning by limiting dilution to ensure stability and monoclonality. Production of MAbs from hybridomas was done in CELLine 1000 bioreactors (Wheaton). Antibodies were purified from supernatant fluids using affinity chromatography and recombinant protein A (rProtein A) Sepharose Fast Flow resin (GE Healthcare).

### Quantitative antigen capture ELISA.

Microtiter plates were coated overnight with MAb 2DA6 (10 µg/ml) in coating buffer (100 mM carbonate, pH 9.6), washed with PBS-Tween (PBS containing 0.05% Tween 20), and blocked for 60 min at 37°C with blocking buffer (PBS containing 0.5% Tween and 5% [wt/vol] powdered milk). Samples of purified mannan (starting concentration of 20 µg/ml) or hot citrate extracts from fungal cultures or infected or control tissue were serially diluted in blocking buffer and incubated for 60 min at 37°C with the MAb-coated wells. Plates were washed with blocking buffer, incubated for 60 min at 37°C with horseradish peroxidase-labeled MAb 2DA6 (2 µg/ml) diluted in blocking buffer, washed with PBS-Tween, and then incubated with tetramethylbenzidine substrate (Kirkegaard & Perry Laboratories, Inc., Gaithersburg, MD). The reaction was stopped after 30 min with a solution of 1 M H_3_PO_4_, and plates were read at an optical density of 450 nm (OD_450_). The dilution of purified mannan or sample extract that produced an OD_450_ of 0.5 in a log-log plot of OD_450_ versus dilution or nanograms of mannan per milliliter was calculated as the endpoint. The endpoint was set at an OD_450_ of 0.5 in order to have a conservative determination of the titer and the assay limit of detection; an OD_450_ of 0.5 is 18 standard deviations above the average background of the MAb 2DA6 antigen capture ELISA (the average background value for wells containing buffer but lacking antigen [*n =* 179] was 0.079, with a standard deviation of 0.023). Depending on the experimental design, results were reported as the reciprocal of the sample dilution at the endpoint (titer) or as the minimal concentration of purified mannan that produced the endpoint OD (limit of detection).

### Lateral flow immunoassay.

A Fusion 5 (GE Healthcare Life Sciences) sample/conjugate pad was pretreated with 0.01 mM borate buffer–0.25% Triton X-100 and dried for 1 h. The test (MAb 2DA6) and control (goat anti-mouse Ig; SouthernBiotech) lines were sprayed onto a Hi-Flow Plus HF120 nitrocellulose membrane (EMD Millipore) at 1 mg/ml and 1 µl/cm using a BioDot XYZ3050 system. The prepared membranes and an absorbent wicking pad (Millipore CFSP203000) were overlapped, assembled using an adhesive backing card, and then cut into 4-mm-wide test strips.

MAb 2DA6 was passively absorbed to 40-nm-diameter colloidal gold particles (DCN Diagnostics) and concentrated to an OD_540_ of 10. The MAb-gold conjugate (5 µl) was applied to the sample/conjugate pad prior to application of citrate extracts of fungal cultures and tissue samples (20 to 40 µl). Dipsticks were then placed vertically into a microtiter well containing 150 µl PBS–1% casein. After 15 min, assays were evaluated visually and digital images were captured.

### Bioinformatics analysis for presence of enzymes involved in synthesis of α-1,6-linked mannose backbone.

A BLASTP search was performed against the NCBI nonredundant protein database (accessed 20 November 2017), which includes all nonredundant GenBank CDS translations as well as all PDB, Swiss-Prot, PIR, and PRF sequences. BLASTP algorithm parameters were set to default values. The query sequence was Mnn9p from S. cerevisiae (Uniprot GenBank accession number P39107). The search set was limited to the indicated fungi. The search set was broadened to include the indicated fungal genus in cases where the genome of a selected fungal species had not yet been sequenced.

### Periodate oxidation and protease digestion.

Periodate oxidation was performed as described previously (26, 27). Briefly, purified mannan (1 mg/ml) was combined with an equal volume of 40 mM sodium meta-periodate (or with water for the mock-treated samples) for 1 h at 4°C (26). Samples were then dialyzed against water to remove excess periodate and any formaldehyde formed during the reaction, followed by reductive amination with an equal volume of 2% (wt/vol) glycine to block the aldehydes. Protease digestion was performed by incubating purified mannan (0.9 mg/ml) with proteinase K (1 mg/ml) for 1 h at 55°C. Samples were then boiled for 10 min for heat inactivation of the proteinase K. Mock-digested mannan was prepared in an identical manner except that water was used in place of proteinase K.

## References

[1] Vos T, Flaxman AD, Naghavi M, Lozano R, Michaud C, Ezzati M, Shibuya K, Salomon JA, Abdalla S, Aboyans V, Abraham J, Ackerman I, Aggarwal R, Ahn SY, Ali MK, Alvarado M, Anderson HR, Anderson LM, Andrews KG, Atkinson C, Baddour LM, Bahalim AN, Barker-Collo S, Barrero LH, Bartels DH, Basáñez MG, Baxter A, Bell ML, Benjamin EJ, Bennett D, Bernabé E, Bhalla K, Bhandari B, Bikbov B, Bin Abdulhak A, Birbeck G, Black JA, Blencowe H, Blore JD, Blyth F, Bolliger I, Bonaventure A, Boufous S, Bourne R, Boussinesq M, Braithwaite T, Brayne C, Bridgett L, Brooker S, et al. 2012 Years lived with disability (YLDs) for 1160 sequelae of 289 diseases and injuries 1990–2010: a systematic analysis for the Global Burden of Disease Study 2010. Lancet 380:2163–2196. doi:10.1016/S0140-6736(12)61729-2. 23245607 PMC6350784

[2] Sobel JD 2007 Vulvovaginal candidosis. Lancet 369:1961–1971. doi:10.1016/S0140-6736(07)60917-9. 17560449

[3] Brown GD, Denning DW, Gow NA, Levitz SM, Netea MG, White TC 2012 Hidden killers: human fungal infections. Sci Transl Med 4:165rv13. doi:10.1126/scitranslmed.3004404. 23253612

[4] Fisher MC, Henk DA, Briggs CJ, Brownstein JS, Madoff LC, McCraw SL, Gurr SJ 2012 Emerging fungal threats to animal, plant and ecosystem health. Nature 484:186–194. doi:10.1038/nature10947. 22498624 PMC3821985

[5] Andersen B, Frisvad JC, Søndergaard I, Rasmussen IS, Larsen LS 2011 Associations between fungal species and water-damaged building materials. Appl Environ Microbiol 77:4180–4188. doi:10.1128/AEM.02513-10. 21531835 PMC3131638

[6] Thornton CR, Wills OE 2015 Immunodetection of fungal and oomycete pathogens: established and emerging threats to human health, animal welfare and global food security. Crit Rev Microbiol 41:27–51. doi:10.3109/1040841X.2013.788995. 23734714

[7] Vidal JE, Boulware DR 2015 Lateral flow assay for cryptococcal antigen: an important advance to improve the continuum of HIV care and reduce cryptococcal meningitis-related mortality. Rev Inst Med Trop Sao Paulo 57(Suppl 19):38–45. doi:10.1590/S0036-46652015000700008. 26465368 PMC4711197

[8] Walsh TJ, Anaissie EJ, Denning DW, Herbrecht R, Kontoyiannis DP, Marr KA, Morrison VA, Segal BH, Steinbach WJ, Stevens DA, van Burik JA, Wingard JR, Patterson TF; Infectious Diseases Society of America 2008 Treatment of aspergillosis: clinical practice guidelines of the Infectious Diseases Society of America. Clin Infect Dis 46:327–360. doi:10.1086/525258. 18177225

[9] Weiner MH, Yount WJ 1976 Mannan antigenemia in the diagnosis of invasive Candida infections. J Clin Invest 58:1045–1053. doi:10.1172/JCI108555. 993329 PMC333270

[10] Reiss E, Lehmann PF 1979 Galactomannan antigenemia in invasive aspergillosis. Infect Immun 25:357–365. 383620 10.1128/iai.25.1.357-365.1979 PMC414460

[11] Wheat LJ, Kohler RB, Tewari RP 1986 Diagnosis of disseminated histoplasmosis by detection of *Histoplasma capsulatum* antigen in serum and urine specimens. N Engl J Med 314:83–88. doi:10.1056/NEJM198601093140205. 3941695

[12] Strahl-Bolsinger S, Gentzsch M, Tanner W 1999 Protein O-mannosylation. Biochim Biophys Acta 1426:297–307. doi:10.1016/S0304-4165(98)00131-7. 9878797

[13] Peat S, Whelan WJ, Edwards TE 1961 Polysaccharides of baker’s yeast. IV. Mannan. J Chem Soc 1:29–34.

[14] Herscovics A, Orlean P 1993 Glycoprotein biosynthesis in yeast. FASEB J 7:540–550. doi:10.1096/fasebj.7.6.8472892. 8472892

[15] Ballou CE 1974 Some aspects of the structure, immunochemistry, and genetic control of yeast mannans. Adv Enzymol Relat Areas Mol Biol 40:239–270. doi:10.1002/9780470122853.ch6. 4599414

[16] Azuma I, Kanetsuna F, Tanaka Y, Yamamura Y, Carbonell LM 1974 Chemical and immunological properties of galactomannans obtained from *Histoplasma dubosii*, *Histoplasma capsulatum*, *Paracoccidioides brasiliensis* and *Blastomyces dermatitidis*. Mycopathol Mycol Appl 54:111–125. 4279334 10.1007/BF02055979

[17] Latgé JP, Kobayashi H, Debeaupuis JP, Diaquin M, Sarfati J, Wieruszeski JM, Parra E, Bouchara JP, Fournet B 1994 Chemical and immunological characterization of the extracellular galactomannan of *Aspergillus fumigatus*. Infect Immun 62:5424–5433. 7960122 10.1128/iai.62.12.5424-5433.1994 PMC303284

[18] Miyazaki T, Yadomae T, Yamada H, Hayashi O, Suzuki I, Ohshima Y 1980 Immunochemical examination of the polysaccharides of mucorales, p 81–94. *In* Sandford PA, Matsuda K (ed), Fungal polysaccharides, Symposium Series No 126. American Chemical Society, Washington, DC.

[19] Kobayashi H, Shibata N, Mitobe H, Ohkubo Y, Suzuki S 1989 Structural study of phosphomannan of yeast-form cells of *Candida albicans* J-1012 strain with special reference to application of mild acetolysis. Arch Biochem Biophys 272:364–375. doi:10.1016/0003-9861(89)90230-0. 2665649

[20] Jungmann J, Munro S 1998 Multi-protein complexes in the cis Golgi of Saccharomyces cerevisiae with alpha-1,6-mannosyltransferase activity. EMBO J 17:423–434. doi:10.1093/emboj/17.2.423. 9430634 PMC1170393

[21] Ballou DL 1975 Genetic control of yeast mannan structure: mapping genes mnn2 and mnn4 in *Saccharomyces cerevisiae*. J Bacteriol 123:616–619. 1097420 10.1128/jb.123.2.616-619.1975 PMC235767

[22] Gopal PK, Ballou CE 1987 Regulation of the protein glycosylation pathway in yeast: structural control of N-linked oligosaccharide elongation. Proc Natl Acad Sci U S A 84:8824–8828. doi:10.1073/pnas.84.24.8824. 3321055 PMC299643

[23] Jungmann J, Rayner JC, Munro S 1999 The *Saccharomyces cerevisiae* protein Mnn10p/Bed1p is a subunit of a Golgi mannosyltransferase complex. J Biol Chem 274:6579–6585. doi:10.1074/jbc.274.10.6579. 10037752

[24] Raschke WC, Kern KA, Antalis C, Ballou CE 1973 Genetic control of yeast mannan structure. Isolation and characterization of mannan mutants. J Biol Chem 248:4660–4666. 4578088

[25] Ballou CE, Kern KA, Raschke WC 1973 Genetic control of yeast mannan structure. Complementation studies and properties of mannan mutants. J Biol Chem 248:4667–4671. 4124122

[26] Hay GW, Lewis BA, Smith F 1965 Periodate oxidation of polysaccharides: general procedures. Methods Carbohydr Chem 5:357–360.

[27] Woodward MP, Young WW, Bloodgood RA 1985 Detection of monoclonal antibodies specific for carbohydrate epitopes using periodate oxidation. J Immunol Methods 78:143–153. doi:10.1016/0022-1759(85)90337-0. 2580026

[28] Bobbitt JM 1956 Periodate oxidation of carbohydrates. Adv Carbohydr Chem 48:1–41. doi:10.1016/S0096-5332(08)60115-0. 13469627

[29] Warkentien T, Rodriguez C, Lloyd B, Wells J, Weintrob A, Dunne JR, Ganesan A, Li P, Bradley W, Gaskins LJ, Seillier-Moiseiwitsch F, Murray CK, Millar EV, Keenan B, Paolino K, Fleming M, Hospenthal DR, Wortmann GW, Landrum ML, Kortepeter MG, Tribble DR; Infectious Disease Clinical Research Program Trauma Infectious Disease Outcomes Study Group 2012 Invasive mold infections following combat-related injuries. Clin Infect Dis 55:1441–1449. doi:10.1093/cid/cis749. 23042971 PMC3657499

[30] Warkentien TE, Shaikh F, Weintrob AC, Rodriguez CJ, Murray CK, Lloyd BA, Ganesan A, Aggarwal D, Carson ML, Tribble DR; Infectious Disease Clinical Research Program Trauma Infectious Disease Outcomes Study Group 2015 Impact of Mucorales and other invasive molds on clinical outcomes of polymicrobial traumatic wound infections. J Clin Microbiol 53:2262–2270. doi:10.1128/JCM.00835-15. 25972413 PMC4473188

[31] Bishop CT, Perry MB, Blank F, Cooper FP 1965 The water-soluble polysaccharides of dermatophytes IV. Galactomannans I from *Trichophyton granulosum*, *Trichophyton interdigitale*, *Microsporum quinckeanum*, *Trichophyton rubrum*, and *Trichophyton schönleinii*. Can J Chem 43:30–39. doi:10.1139/v65-005.

[32] Bishop CT, Perry MB, Blank F 1966 The water-soluble polysaccharides of dermatophytes V. Galactomannans II from *Trichophyton granulosum*, *Trichophyton interdigitale*, *Microsporum quinckeanum*, *Trichophyton rubrum*, and *Trichophyton schönleinii*. Can J Chem 44:2291–2297. doi:10.1139/v66-344.

[33] Ikuta K, Shibata N, Blake JS, Dahl MV, Nelson RD, Hisamichi K, Kobayashi H, Suzuki S, Okawa Y 1997 NMR study of the galactomannans of *Trichophyton mentagrophytes* and *Trichophyton rubrum*. Biochem J 323:297–305. doi:10.1042/bj3230297. 9173896 PMC1218309

[34] Carlson P, Richardson M, Paavonen J 2000 Evaluation of the Oricult-N dipslide for laboratory diagnosis of vaginal candidiasis. J Clin Microbiol 38:1063–1065. 10698997 10.1128/jcm.38.3.1063-1065.2000 PMC86339

[35] Reiss E 1986 Molecular immunology of mycotic and actinomycotic infections. Elsevier Science, New York, NY.

[36] Jones GH, Ballou CE 1969 Studies on the structure of yeast mannan. II. Mode of action of the Arthrobacter alpha-mannosidase on yeast mannan. J Biol Chem 244:1052–1059. 5814027

[37] Maitra SK, Ballou CE 1976 Characterization of a mannan-like oligosaccharide from *Mycobacterium smegmatis*. Biochem Biophys Res Commun 73:1101–1108. doi:10.1016/0006-291X(76)90236-9. 15625887

[38] Yokoyama K, Ballou CE 1989 Synthesis of alpha 1-6-mannooligosaccharides in Mycobacterium smegmatis. Function of beta-mannosylphosphoryldecaprenol as the mannosyl donor. J Biol Chem 264:21621–21628. 2480954

[39] Mishra AK, Alderwick LJ, Rittmann D, Wang C, Bhatt A, Jacobs WR, Takayama K, Eggeling L, Besra GS 2008 Identification of a novel alpha(1→6) mannopyranosyltransferase MptB from Corynebacterium glutamicum by deletion of a conserved gene, NCgl1505, affords a lipomannan- and lipoarabinomannan-deficient mutant. Mol Microbiol 68:1595–1613. doi:10.1111/j.1365-2958.2008.06265.x. 18452585 PMC2440535

[40] Angala SK, Palčeková Z, Belardinelli JM, Jackson M 2018 Covalent modifications of polysaccharides in mycobacteria. Nat Chem Biol 14:193–198. doi:10.1038/nchembio.2571. 29443974 PMC5831173

[41] Blehert DS, Hicks AC, Behr M, Meteyer CU, Berlowski-Zier BM, Buckles EL, Coleman JT, Darling SR, Gargas A, Niver R, Okoniewski JC, Rudd RJ, Stone WB 2009 Bat white-nose syndrome: an emerging fungal pathogen? Science 323:227. doi:10.1126/science.1163874. 18974316

[42] Lorch JM, Meteyer CU, Behr MJ, Boyles JG, Cryan PM, Hicks AC, Ballmann AE, Coleman JT, Redell DN, Reeder DM, Blehert DS 2011 Experimental infection of bats with *Geomyces destructans* causes white-nose syndrome. Nature 480:376–378. doi:10.1038/nature10590. 22031324

[43] Ma L, Chen Z, Huang DW, Kutty G, Ishihara M, Wang H, Abouelleil A, Bishop L, Davey E, Deng R, Deng X, Fan L, Fantoni G, Fitzgerald M, Gogineni E, Goldberg JM, Handley G, Hu X, Huber C, Jiao X, Jones K, Levin JZ, Liu Y, Macdonald P, Melnikov A, Raley C, Sassi M, Sherman BT, Song X, Sykes S, Tran B, Walsh L, Xia Y, Yang J, Young S, Zeng Q, Zheng X, Stephens R, Nusbaum C, Birren BW, Azadi P, Lempicki RA, Cuomo CA, Kovacs JA 2016 Genome analysis of three Pneumocystis species reveals adaptation mechanisms to life exclusively in mammalian hosts. Nat Commun 7:10740. 26899007 10.1038/ncomms10740 PMC4764891

[44] Krajicek BJ, Kottom TJ, Villegas L, Limper AH 2010 Characterization of the PcCdc42 small G protein from *Pneumocystis carinii*, which interacts with the PcSte20 life cycle regulatory kinase. Am J Physiol Lung Cell Mol Physiol 298:L252–L260. doi:10.1152/ajplung.00191.2009. 19915161 PMC2822561

[45] Dubois M, Gilles KA, Hamilton JK, Rebers PA, Smith F 1956 Colorimetric method for determination of surgars and related substances. Anal Chem 28:350–356. doi:10.1021/ac60111a017.

[46] Hasenclever HF, Mitchell WO 1960 Antigenic relationships of *Torulopsis glabrata* and seven species of the genus *Candida*. J Bacteriol 79:677–681. 13851946 10.1128/jb.79.5.677-681.1960 PMC278757

[47] Osterland CK, Miller EJ, Karakawa WW, Krause RM 1966 Characteristics of streptococcal group-specific antibody isolated from hyperimmune rabbits. J Exp Med 123:599–614. doi:10.1084/jem.123.4.599. 4160396 PMC2180455

[48] Marusich MF 1988 Efficient hybridoma production using previously frozen splenocytes. J Immunol Methods 114:155–159. doi:10.1016/0022-1759(88)90167-6. 3183388

