# Supplementary material for: Conservation of Mannan Synthesis in Fungi of the Zygomycota and Ascomycota Reveals a Broad Diagnostic Target
Source: mSphere. 2018 May 2;3(3):e00094-18. doi: 10.1128/mSphere.00094-18 (PMC5932377; doi:10.1128/mSphere.00094-18)
Supplement: TABLE S1 [file sph003182538st1.pdf]

Table S1. Predicted reactivity of mAb 2DA6 with major fungal pathogens in plant pathology[a]

Fungus	Disease	Phylum	Mnn9p homology		Predicted reactivity with mAb 2DA6[c]
			Accession #	Homology	
Magnaporthe oryzae	Rice blast	Ascomycota	XP_003718954.1	2e-119	Yes
Botrytis cinerea	Necrotropic fungus; many hosts	Ascomycota	XP_001556212.1	6e-113	Yes
Puccinia spp	Rust diseases on wheat	Basidiomycota	None	None	No
Fusarium graminearum	Head blight of wheat	Ascomycota	OBS18092.1	2e-119	Yes
Fusarium oxysporum	Fusarium wilt; many hosts	Ascomycota	EXA49808.1	3e-118	Yes
Blumeria graminis	Powdery mildew of grasses	Ascomycota	EPQ65832.1	9e-112	Yes
Mycosphaerella graminicola	*Septoria tritici* blight of wheat	Ascomycota	XP_003857008.1	3e-127	Yes
Colletotrichum spp.	Multiple diseases; multiple hosts	Ascomycota	OLN88651.1	9e-119	Yes
Ustilago maydis	Corn smut	Basidiomycota	None	None	No
Melampsora lini	Flax rust	Basidiomycota	ND[b]	ND	No

[a]Fungi selected from: Dean R, Van Kan JA, Pretorius ZA, Hammond-Kosack KE, Di Pietro A, Spanu PD, Rudd JJ, Dickman M, Kahmann R, Ellis J, Foster GD. 2012. The Top 10 fungal pathogens in molecular plant pathology. Mol Plant Pathol 13:414-430.

[b]Not determined; too few sequences in NCBI database for homology search.

[c]Reactivity with mAb 2DA6 is predicted when a fungus is both a member of the Zygomycota or Ascomycota and there is a Mnn9p homologue. If the fungus is a member of the Zygomycota or Ascomycota but there is insufficient information in the NCBI database to assess Mnn9p homology, predicted reactivity is considered “probable.” If the fungus is a member of the Zygomycota or the Ascomycota and there is no Mnn9p homologue, predicted reactivity is considered “indeterminate.” In cases of indeterminate reactivity, discrepancy must be resolved by direct experimentation.
