# Supplementary material for: Conservation of Mannan Synthesis in Fungi of the Zygomycota and Ascomycota Reveals a Broad Diagnostic Target
Source: mSphere. 2018 May 2;3(3):e00094-18. doi: 10.1128/mSphere.00094-18 (PMC5932377; doi:10.1128/mSphere.00094-18)
Supplement: TABLE S2 [file sph003182538st2.pdf]

Table S2. Predicted reactivity of mAb 2DA6 with common fungal causes of invasive fungal infection in humans[a]

Fungus	Disease	Phylum	Mnn9p homology		Predicted reactivity with mAb 2DA6[d]
			Accession #	Homology	
Cryptococcus neoformans	Cryptococcosis	Basidiomycota	None	None	No
Candida albicans	Invasive candidiasis	Ascomycota	XP_716624.1	1e-149	Yes
Pneumocystis jirovecii	Pneumocystis pneumonia	Ascomycota	None	None	Indeterminate
Aspergillus spp.	Aspergillosis	Ascomycota	XP_001273073.1	4e-126[b]	Yes
Coccidioides immitis	Coccidioidomycosis	Ascomycota	XP_001246370.1	9e-122	Yes
Histoplasma capsulatum	Histoplasmosis	Ascomycota	EGC49211.1	2e-120	Yes
Rhizopus and *Mucor* spp.	Mucormycosis	Zygomycota	CEG79707.1	6e-70[c]	Yes
Talaromyces marneffei	Penicilliosis	Ascomycota	XP_002143795.1	1e-120	Yes
Paracoccidioides brasiliensis	Paracoccidioido-mycosis	Ascomycota	XP_010758816.1	6e-117	Yes
Blastomyces dermatitidis	Blastomycosis	Ascomycota	EEQ87264.1	4e-121	Yes

[a]Fungi selected from: Brown GD, Denning DW, Gow NA, Levitz SM, Netea MG, White TC. 2012. Hidden killers: human fungal infections. Sci Transl Med 4:165rv113.

[b]Results are shown for a search of the genus *Aspergillus*.

[c]The protein sequence with the greatest similarity (that shown) is from *Rhizopus*.

[d]Reactivity with mAb 2DA6 is predicted when a fungus is both a member of the Zygomycota or Ascomycota and there is a Mnn9p homologue. If the fungus is a member of the Zygomycota or Ascomycota but there is insufficient information in the database to assess Mnn9p homology, predicted reactivity is considered “probable.” If the fungus is a member of the Zygomycota or the Ascomycota and there is no Mnn9p homologue, predicted reactivity is considered “indeterminate.” In cases of indeterminate reactivity, discrepancy must be resolved by direct experimentation. In the case of *P. carinii*, direct experimentation did, in fact, determine that the fungus does not make a mannan with mAb 2DA6 reactivity (manuscript Fig. 4).
