# Supplementary material for: Conservation of Mannan Synthesis in Fungi of the Zygomycota and Ascomycota Reveals a Broad Diagnostic Target
Source: mSphere. 2018 May 2;3(3):e00094-18. doi: 10.1128/mSphere.00094-18 (PMC5932377; doi:10.1128/mSphere.00094-18)
Supplement: TABLE S3 [file sph003182538st3.pdf]

Table S3. Predicted reactivity of mAb 2DA6 with significant fungal causes of skin, hair, nail, eye, cutaneous and subcutaneous fungal infections in humans[a]

Fungus	Disease	Phylum	Mnn9p homology		Predicted reactivity with mAb 2DA6[c]
			Accession #	Homology	
Trichophyton spp.	Dermatophytosis	Ascomycota	XP_003238615.1	9e-119	Yes
Microsporum spp.	Dermatophytosis	Ascomycota	XP_002850066.1	4e-115	Yes
Epidermophyton floccosum	Dermatophytosis	Ascomycota	ND[b]	ND	Probable
Malassezia spp.	Pityriasis versicolor	Basidiomycota	None	None	No
Candida albicans	Candida vaginitis	Ascomycota	XP_716624.1	1e-149	Yes
Fusarium spp.	Fungal keratitis	Ascomycota	XP_003051726.1	7e-120	Yes
Sporothrix schenckii	Sporotrichosis	Ascomycota	XP_016587079.1	3e-112	Yes
Fonsecaea spp.	Chromoblastomycosis	Ascomycota	XP_016634636.1	1e-119	Yes
Madurella mycetomatis	Eumycetoma	Ascomycota	KXX81953.1	4e-67	Yes
Bipolaris spp.	Subcutaneous phaeohyphomycosis	Ascomycota	XP_007705235.1	1e-117	Yes

[a]Fungi selected from chapters in: Bennett JE, Dolin R, Blaser MJ. 2015. Mandell, Douglas and Bennett's principles and practice of infectious diseases, 8th ed. Elsevier Saunders.

[b]Not determined; too few sequences in NCBI database for homology search.

[c]Reactivity with mAb 2DA6 is predicted when a fungus is both a member of the Zygomycota or Ascomycota and there is a Mnn9p homologue. If the fungus is a member of the Zygomycota or Ascomycota but there is insufficient information in the NCBI database to assess Mnn9p homology, predicted reactivity is considered “probable.” If the fungus is a member of the Zygomycota or the Ascomycota and there is no Mnn9p homologue, predicted reactivity is considered “indeterminate.” In cases of indeterminate reactivity, discrepancy must be resolved by direct experimentation.
