# Supplementary material for: Conservation of Mannan Synthesis in Fungi of the Zygomycota and Ascomycota Reveals a Broad Diagnostic Target
Source: mSphere. 2018 May 2;3(3):e00094-18. doi: 10.1128/mSphere.00094-18 (PMC5932377; doi:10.1128/mSphere.00094-18)
Supplement: TABLE S4 [file sph003182538st4.pdf]

Table S4. Predicted reactivity of mAb 2DA6 with major fungal causes of potential biodiversity loss[a]

Fungus	Disease	Phylum	Mnn9p homology		Predicted reactivity with mAb 2DA6[d]
			Accession #	Homology	
Batrachochytrium dendrobatidis	Chytridiomycosis in amphibians	Chytridiomycota	None	None	No
Ophidiomyces ophiodiicola	Snake fungal disease	Ascomycota	ND[b]	ND	Probable
Pseudogymnoascus destructans	Bat white-nose disease	Ascomycota	OAF58468.1	3e-118	Yes
Aspergillus sydowii	Sea fan aspergillosis	Ascomycota	XP_001273073.1[c]	4e-126	Yes
Nosema spp.	Bee colony collapse disorder	Microsporidia	None	None	No
Fusarium solani species complex	Hatch failure in sea turtles	Ascomycota	XP_003051726.1	3e-121	Yes

[a]Fungi selected in part from: Fisher MC, Henk DA, Briggs CJ, Brownstein JS, Madoff LC, McCraw SL, Gurr SJ. 2012. Emerging fungal threats to animal, plant and ecosystem health. Nature 484:186-194.

[b]Not determined; too few sequences in NCBI database for homology search.

[c] Results are shown for a search of the genus *Aspergillus*.

[d]Reactivity with mAb 2DA6 is predicted when a fungus is both a member of the Zygomycota or Ascomycota and there is a Mnn9p homologue. If the fungus is a member of the Zygomycota or Ascomycota but there is insufficient information in the NCBI database to assess Mnn9p homology, predicted reactivity is considered “probable.” If the fungus is a member of the Zygomycota or the Ascomycota and there is no Mnn9 homologue, predicted reactivity is considered “indeterminate.” In cases of indeterminate reactivity, discrepancy must be resolved by direct experimentation.
