# Supplementary material for: Conservation of Mannan Synthesis in Fungi of the Zygomycota and Ascomycota Reveals a Broad Diagnostic Target
Source: mSphere. 2018 May 2;3(3):e00094-18. doi: 10.1128/mSphere.00094-18 (PMC5932377; doi:10.1128/mSphere.00094-18)
Supplement: TABLE S5 [file sph003182538st5.pdf]

Table S5. Predicted reactivity of mAb 2DA6 with agents of fungal disease-driven and regional extirpation events across animal and plant taxa[a]

Fungus	Disease	Phylum	Mnn9p homology		Predicted reactivity with mAb 2DA6[d]
			Accession #	Homology	
Batrachochytrium dendrobatidis	Amphibian extinction/extirpation	Chytridomycota	None	None	No
Pseudogymnoascus destructans	Bat extirpation	Ascomycota	OAF58468.1	3e-118	Yes
Steinhausia spp.	Hawaiian tree snail extinction	Microsporidia	ND[b]	ND	No
Cryphonectria parasitica	Chestnut tree extirpation	Ascomycota	ND	ND	Probable
Ophiostoma spp.	Elm tree extirpation	Ascomycota	EPE06315.1[c]	1e-109	Yes
Fusarium circinatum	Monterey pine extirpation	Ascomycota	XP_003051726.1[c]	7e-120	Yes
Pestalotiopsis spp.	Florida torreya extinction (~99%)	Ascomycota	XP_007827205.1[c]	1e-114	Yes

[a]Fungi selected from: Fisher MC, Henk DA, Briggs CJ, Brownstein JS, Madoff LC, McCraw SL, Gurr SJ. 2012. Emerging fungal threats to animal, plant and ecosystem health. Nature 484:186-194.

[b]Not determined; too few sequences in NCBI database for homology search.

[c]Search done at genus level.

[d]Reactivity with mAb 2DA6 is predicted when a fungus is both a member of the Zygomycota or Ascomycota and there is a Mnn9p homologue. If the fungus is a member of the Zygomycota or Ascomycota but there is insufficient information in the NCBI database to assess Mnn9p homology, predicted reactivity is considered “probable.” If the fungus is a member of the Zygomycota or the Ascomycota and there is no Mnn9p homologue, predicted reactivity is considered “indeterminate.” In cases of indeterminate reactivity, discrepancy must be resolved by direct experimentation.
