# Supplementary material for: Conservation of Mannan Synthesis in Fungi of the Zygomycota and Ascomycota Reveals a Broad Diagnostic Target
Source: mSphere. 2018 May 2;3(3):e00094-18. doi: 10.1128/mSphere.00094-18 (PMC5932377; doi:10.1128/mSphere.00094-18)
Supplement: TABLE S6 [file sph003182538st6.pdf]

Table S6. Predicted reactivity of mAb 2DA6 with selected fungal agents of food and agricultural spoilage[a]

Fungus	Disease	Phylum	Mnn9p homology		Predicted reactivity with mAb 2DA6[b]
			Accession #	Homology	
Rhizopus spp.	Vegetables and fruits; bread mold	Zygomycota	CEG79707.1	4e-70	Yes
Botrytis cinerea	Soft fruits	Ascomycota	XP_001556212.1	6e-113	Yes
Cladosporium spp.	Grains	Ascomycota	None	None	Indeterminate
Alternaria spp.	Grains, fruit	Ascomycota	XP_018382478.1	5e-119	Yes
Fusarium spp.	Grains	Ascomycota	XP_003051726.1	7e-120	Yes
Penicillium spp.	Grains, fruit	Ascomycota	CEJ56560.1	3e-125	Yes
Aspergillus spp.	Grains	Ascomycota	XP_001273073.1	4e-126	Yes

[a] Fungi selected from: Lacey J. 1989. Pre- and post-harvest ecology of fungi causing spoilage of foods and other stored products. Soc Appl Bacteriol Symp Ser 18:11S-25S and Filtenborg O, Frisvad JC, Thrane U. 1996. Moulds in food spoilage. Int J Food Microbiol 33:85-102..

[b] Reactivity with mAb 2DA6 is predicted when a fungus is both a member of the Zygomycota or Ascomycota and there is a Mnn9p homologue. If the fungus is a member of the Zygomycota or Ascomycota but there is insufficient information in the NCBI database to assess Mnn9p homology, predicted reactivity is considered “probable.” If the fungus is a member of the Zygomycota or the Ascomycota and there is no Mnn9p homologue, predicted reactivity is considered “indeterminate.” In cases of indeterminate reactivity, discrepancy must be resolved by direct experimentation.
