# Supplementary material for: Conservation of Mannan Synthesis in Fungi of the Zygomycota and Ascomycota Reveals a Broad Diagnostic Target
Source: mSphere. 2018 May 2;3(3):e00094-18. doi: 10.1128/mSphere.00094-18 (PMC5932377; doi:10.1128/mSphere.00094-18)
Supplement: TABLE S7 [file sph003182538st7.pdf]

Table S7. Predicted reactivity of mAb 2DA6 with fungal genera commonly found on water-damaged building materials[a]

Fungus	Phylum	Mnn9p homology		Predicted reactivity with mAb 2DA6[c]
		Accession #	Homology	
Penicillium spp.	Ascomycota	CEJ56560.1	3e-125	Yes
Aspergillus spp.	Ascomycota	XP_001273073.1	4e-126	Yes
Chaetomium spp.	Ascomycota	XP_006696111.1	2e-119	Yes
Acremonium spp.	Ascomycota	None	None	Indeterminate
Ulocladium spp.	Ascomycota	ND[b]	ND	Probable
Cladosporium spp.	Ascomycota	None	None	Indeterminate
Mucor spp.	Zygomycota	EPB85583.1	9e-69	Yes
Trichoderma spp.	Ascomycota	OTA01784.1	7e-122	Yes
Alternaria spp.	Ascomycota	XP_018382478.1	5e-119	Yes
Sporothrix spp.	Ascomycota	XP_016587079.1	6e-112	Yes

[a]Fungi selected from: Andersen B, Frisvad JC, Søndergaard I, Rasmussen IS, Larsen LS. 2011. Associations between fungal species and water-damaged building materials. Appl Environ Microbiol 77:4180-4188.

[b]Not determined; too few sequences in NCBI database for homology search.

[c]Reactivity with mAb 2DA6 is predicted when a fungus is both a member of the Zygomycota or Ascomycota and there is a Mnn9p homologue. If the fungus is a member of the Zygomycota or Ascomycota but there is insufficient information in the NCBI database to assess Mnn9p homology, predicted reactivity is considered “probable.” If the fungus is a member of the Zygomycota or the Ascomycota and there is no Mnn9p homologue, predicted reactivity is considered “indeterminate.” In cases of indeterminate reactivity, discrepancy must be resolved by direct experimentation.
